# Comparison of Propofol and Dexmedetomidine Infused Overnight to Treat Hyperactive and Mixed ICU Delirium: A Prospective Randomised Controlled Clinical Trial

**DOI:** 10.3390/jcm14124348

**Published:** 2025-06-18

**Authors:** Stefan Zimmermann, Alexa Hollinger, Rita Achermann, Stefanie von Felten, Raoul Sutter, Stephan Rüegg, Salim Abdelhamid, Simon Glatz, Luzius A. Steiner, Martin Siegemund

**Affiliations:** 1 Intensive Care Unit, University Hospital Basel, Spitalstrasse 21, 4031 Basel, Switzerland; stefanurs.zimmermann@usb.ch (S.Z.); rita.achermann@usb.ch (R.A.); raoul.sutter@usb.ch (R.S.); salim.abdelhamid@gmail.ch (S.A.); simon.glatz@usb.ch (S.G.); martin.siegemund@usb.ch (M.S.); 2 Medical Faculty, University of Basel, 4056 Basel, Switzerland; stephan.rueegg@usb.ch (S.R.); luzius.steiner@usb.ch (L.A.S.); 3 Department of Clinical Research, University of Basel, University Hospital Basel, 4031 Basel, Switzerland; stefanie.vonfelten@uzh.ch; 4 Epidemiology, Biostatistics and Prevention Institute (EBPI), University of Zurich, 8001 Zurich, Switzerland; 5 Department for Clinical Neurophysiology, Epilepsy and Movement Disorders, University Hospital Basel, 4031 Basel, Switzerland; 6 Department of Anesthesia, Prehospital Emergency Medicine and Pain Therapy, University Hospital Basel, 4031 Basel, Switzerland

**Keywords:** critical care, delirium treatment, intensive care unit, RCT, sedation

## Abstract

Background: Delirium is a frequent yet pathophysiologically still poorly understood complication in the intensive care unit (ICU) and is associated with adverse outcomes for the patients. Currently, guidelines give several recommendations for treating delirium in the ICU, but to date no sufficient drug treatment exists. Dexmedetomidine, primarily used for anesthesia and sedation in ICUs has shown a preventive effect of delirium compared to other sedatives, such as propofol. We hypothesize that overnight administration of dexmedetomidine may prevent and/or shorten the duration of delirium in ICU patients. Methods: The Basel propofol dexmedetomidine (BaProDex) Study was a single-center, prospective, randomized controlled trial. We included adult ICU patients with hyperactive or mixed delirium. Patients with delirium prior to ICU admission, advanced heart block, uncontrolled hypotension, or status epilepticus were excluded. The participants were randomly assigned 1:1 to either receive dexmedetomidine (study group) or propofol (control group) as a continuous infusion overnight. The Intensive Care Delirium Screening Checklist (ICDSC) was applied at least three times per day. Delirium was defined as an ICDSC ≥ 4. The study drug was administered until the end of delirium or ICU discharge. The primary endpoint was the time to delirium episode end, which was analyzed using cumulative incidence curves and a cause specific Cox proportional hazards regression with death as a competing risk. Secondary endpoints included recurrence of delirium until 28 days after ICU discharge, death until day 28, severity of ICU delirium, number of ventilation days, ICU length of stay (LOS) in hours, hospital length of stay in days and survival after three and twelve months after ICU discharge. Due to insufficient recruitment the trial needed to be stopped prematurely. Results: In total, 38 patients were enrolled and randomized in the two groups. The median duration of delirium was shorter in the dexmedetomidine group as compared to the propofol group (ITT: 34 vs. 66 h; PP: 31 vs. 66 h), resulting in a hazard ratio of 1.92 (95% CI 0.89–4.15, *p* = 0.097) in the ITT and 2.95 (95% CI 1.27–6.86, *p* = 0.012) in the PP analysis. In the PP analysis, the 28-day mortality was lower in the dexmedetomidine group (1 vs. 5 deaths) and fewer patients needed ventilation (7 vs. 15 patients). Both ICU and hospital LOS were shorter in the dexmedetomidine group (ICU LOS: median 43 vs. 128 h; hospital LOS: median 12 vs. 22 days). Further, mortality up to three and twelve months was lower in the dexmedetomidine group compared to the propofol group (PP: 2 vs. 8 patients died within twelve months, 2 vs. 7 patients died within three months). The recurrence of delirium until 28 days after ICU discharge and severity of delirium were similar in both groups. Conclusions: Despite premature termination, BaProDex provides preliminary evidence for a reduction in the duration of delirium by nocturnal infusion of dexmedetomidine compared to propofol. Therefore, dexmedetomidine may be considered an option to treat hyperactive or mixed delirium in ICU patients. However, due to the small sample size, the study is rather of exploratory nature due to the premature termination, and we cannot rule out that the observed treatment effect is overly optimistic or by chance.

## 1. Introduction

Delirium is a severe neurocognitive disorder characterized by acute brain dysfunction that results in altered attention and awareness [1,2]. It is usually categorized into three subgroups according to the symptomatic motoric presentation: hyperactive, hypoactive, and mixed type [3]. The disruption of a normal sleep cycle may be the promoter and/or consequence of delirium [4].

Delirium is frequent among patients in the intensive care unit (ICU). A meta-analysis in 2018 showed that the prevalence of delirium in adults in the ICU was 31% (hypoactive 17%, hyperactive 4%, mixed 10%) [3]. It is associated with serious individual and socioeconomic consequences, including increased length of hospital stay, subsequent hospitalization, complications, increased mortality and higher treatment costs [5,6,7]. There are also long-term side effects, such as worse long-term cognitive and executive function with loss of independence and acceleration of dementia [1,8]. As the population, in general, is getting older, the prevalence of age-related syndromes, such as delirium, is likely to increase, thereby gaining even more importance [9].

The latest American guidelines for the treatment of delirium in the ICU do not provide strong recommendations regarding the medical treatment of delirium because of a lack of evidence [10]. Potential medical treatment options, such as haloperidol or atypical antipsychotics are not recommended for routine use due to the lack of evidence and possible negative effects [7,10]. There may be situations where these drugs are beneficial for reducing the symptoms of delirium. However, these drugs do not treat the delirium itself and do not reduce delirium incidence or reduce the adverse effects associated with delirium [7,11]. In some cases, sedation is necessary to reduce hyperactive symptoms and to protect patients from self-inflicted harm [5]. Unfortunately, sedatives should be administered with caution, as they can promote and potentiate acute brain dysfunction and they have negative effects on long-term cognitive function [12].

An alternative to these drugs is dexmedetomidine, a selective alpha-2 adrenergic receptor agonist with anti-sympathetic, anxiolytic, sedative, and analgetic effects, which is already used for sedation in critical care [13]. It acts on mechanisms involving endogenous sleep pathways and induces a sedation, which resembles physiological sleep patterns in electroencephalography (EEG) and may therefore produce a condition comparable to restorative sleep and may help to recover the day/night cycle of patients [5,14,15]. It was further shown that nocturnal low dose administration of dexmedetomidine can reduce the incidence of delirium in critically ill ICU patients [16].

In addition to studies revealing that sedation with dexmedetomidine is superior to propofol in elderly ICU patients to prevent delirium [17], the question of if dexmedetomidine could also be an option in the treatment of delirium is yet poorly examined.

We hypothesized that nocturnal continuous infusions of dexmedetomidine may decrease the duration of delirium compared to propofol by establishing a simulated normal day–night cycle especially in patients with hyperactive or mixed delirium [5].

## 2. Materials and Methods

### 2.1. Trial Design

The Basel ProDex clinical trial was a single-center, parallel group, randomized controlled trial of adult patients suffering from hyperactive or mixed delirium and hospitalized at the ICU of the University Hospital Basel, a Swiss academic tertiary medical care center. The trial was registered on www.ClinicalTrials.gov on 21 June 2016; registration number NCT02807467. The study protocol was previously published [5].

### 2.2. Study Population

All adult patients admitted to our ICU were screened daily for delirium using the Intensive Care Delirium Screening Checklist (ICDSC) routinely used in our ICU (see Section 2.3.2). All patients with current hyperactive or mixed-type delirium who had an ICDSC-Score of ≥4 in two consecutive shifts (shifts determined from 07:00 a.m.–02:59 p.m., 03:00 p.m.–10:59 p.m., and 11:00 p.m.–06:59 a.m.) were considered eligible. Patients were excluded if they were delirious prior to ICU admission, had an egg or soy allergy, hypersensitivity to the active substances, an advanced heart block without pacemaker, bradycardia of any origin, or uncontrolled hypotension. Patients with acute cerebrovascular conditions including ischemic stroke or intracranial hemorrhage or suffering from delirium tremens or substance abuse withdrawal symptoms, or who were in a terminal state were also excluded. Definitions of eligibility criteria are provided in the previously published study protocol [5]. We assigned eligible patients in a 1:1 ratio in one of two groups to receive either dexmedetomidine or propofol using a variance minimization algorithm performed by the Clinical Trial Unit (CTU), Basel. The variance minimization algorithm ensured that the treatment groups were balanced for the potential prognostic factor surgical reason at admission to hospital = cardiac surgery (yes/no). Delirium end was defined as the first ICDSC < 4 followed by a second ICDSC < 4 in the subsequent shift.

### 2.3. Trial Interventions and Assessments

#### 2.3.1. Study Drugs

Prior to the application of the study medication, all patients were treated according to the internal guidelines used in our ICU. First-line sedatives in the acute setting were intravenous haloperidol, followed by oral quetiapine or in the case of pre-existing cerebral dysfunction, trazodone hydrochloride (for Standard Operating Procedure of the University Hospital Basel see Supplementary Materials). After enrolment, patients received a continuous infusion of either dexmedetomidine (concentrated 200 µg/2 mL, Orion Pharma AG, Orion Corporation, Espoo, Finland) or intravenous propofol Lipuro (1%, concentrated 1 g/100 mL, B. Braun Medical AG, Sempach, Switzerland/Fresenius Kabi (Schweiz) AG, Am Mattenhof 4, 6010 Kriens, Switzerland) between 08:00 p.m. and 06:00 a.m. daily. In cases of patient’s delirium being refractory to the first administration of sedatives, the treating physicians were allowed to intravenously administer haloperidol (Haldol, Janssen-Cilag AG, Schaffhausen, Switzerland) as first line rescue therapy or if not sufficient other additional sedatives and neuroleptics (quetiapine, trazodone, lorazepam, midazolam). If an intravenously administered sedation during daytime was needed because of uncontrolled agitation with the risk of self-inflicted injury and/or aggressive behavior, patients received the assigned study drug. All additional sedatives given were recorded. The study medication was administered every night until delirium resolution was perceived or due to early termination because of patient death, patient transfer to a general ward or withdrawal of consent. To assess long-term safety endpoints, we performed a three-month and a twelve-month follow-up of mortality and functionality in daily life using the activities of daily life questionnaire (ADLQ).

#### 2.3.2. Delirium Assessment

To define delirium and assess its length and severity we used the ICDSC, as described above. The depth of sedation and/or level of agitation was assessed using the Richmond Agitation–Sedation Scale (RASS). All assessments were carried out by the treating intensive care nurse expert. Assessments that could not be properly performed, for example, because the patient was unconscious, were marked as incomplete. Scores were assessed at least once during each shift. To assess occurrence of delirium after discharge from the ICU we also analyzed delirium scores evaluated at the general ward (assessed by the Delirium Observation Scale, DOS; assessment in case of suspected delirium). Additional information can be found in the study protocol [5].

#### 2.3.3. Adverse Events

We recorded all routinely collected vital parameters (e.g., heart rate, blood pressure, fluid balance) and laboratory parameters (e.g., blood count, creatinine, blood urea nitrogen, triglycerides) daily to detect possible drug side effects of the study medication, such as arterial hypotension, bradycardia, or the propofol-related infusion syndrome (PRIS).

### 2.4. Study Outcomes

The primary endpoint was the duration of the delirium episode. It was calculated as hours between the first administration of the study drug and the end of the delirium episode, defined as the time of the last ICDSC ≥ 4 followed by two subsequent shifts with ICDSC < 4.

Secondary endpoints were recurrence of delirium until 28 days after ICU discharge, death until day 28, severity of ICU delirium (ICDSC), number of ventilator days, need for rescue sedation (amount of haloperidol, mg), amount of oral Quetiapine, total costs of medication (dexmedetomidine infused + rescue medication or propofol infusion + rescue medication), ICU length of stay in hours, length of hospital stay in days, depth of sedation (RASS), survival after three and twelve months after ICU discharge, and ADLQ before three and twelve months after ICU admission.

### 2.5. Sample Size

Sample size was calculated with a semi-parametric resampling method [18] based on pilot data and simulation. This allowed for accounting non-parametrically for the distribution of delirium duration in the pilot data set and parametrically for the treatment shift. The pilot data set, with a median delirium duration of 96 h, was assumed to represent patients treated with propofol and we expected a reduction in delirium duration with dexmedetomidine compared to propofol of 25% in line with current research results [17]. Based on a Wilcoxon rank sum test, we calculated that 300 patients are required to ensure a power of 80% at a significance level of 5%. With a drop-out rate of 5%, 316 patients should be recruited. More detail on the exact calculation can be found in the study protocol [5].

#### Early Termination of the Study Because of Slow Recruitment

The recruitment started in April 2019. As the enrolment was slower than anticipated, we submitted an amendment in March of 2021 of the study protocol to the local ethics committee and removed “acute cerebrovascular conditions”, “alcohol abuse”, “delirium tremens”, and “substance abuse with experience of acute withdrawal” from the exclusion criteria. In addition, the inclusion criteria were modified, such that only one shift with an ICDSC-Score of ≥4 was needed to be eligible for the study. Despite these changes within the eligibility criteria, we were unable to recruit more patients. Therefore, the study was prematurely terminated in March 2022 with 38 patients instead of the planned 316 patients enrolled. The last patient was enrolled in November 2021, and the last one-year follow up was performed in November 2022.

### 2.6. Statistical Analysis

**Figure 1:**
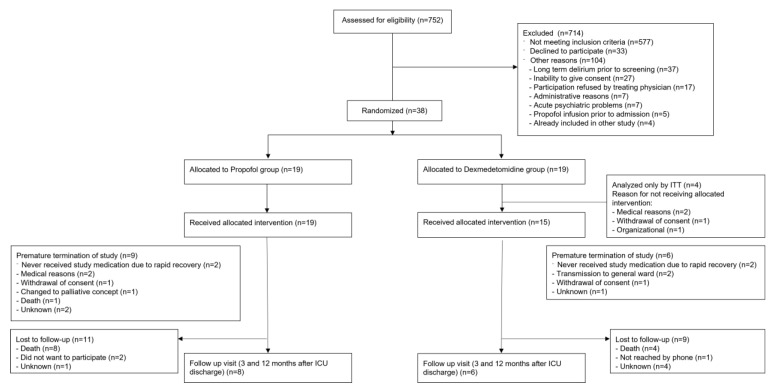
Flow chart.

The primary outcome, time to delirium episode end, was analyzed using a cause-specific Cox proportional hazards regression to estimate the hazard ratio between the two study groups unadjusted and adjusted for confounders cardia surgery (yes/no) and the Simplified Acute Physiology Score II (SAPS II). In the intention to treat analysis, all randomized patients were included, in the per protocol analysis, four patients in the dexmedetomidine group were excluded, as they never received the allocated treatment (Figure 1). Patients who did not receive the treatment because the delirium episode ended prematurely were still included in the per protocol analysis. If the study treatment was interrupted, for example, because the patient had surgery, the patient was still included in the per protocol analysis. In addition, the delirium episode duration was plotted as cumulative incidence using the Gray-method to account for censored delirium episodes with competing risk death, and the median delirium episode duration was calculated.

Secondary outcomes were analyzed descriptively for the patients included in the ITT analysis and for hospital length of stay and mortality for PP analysis. Statistical tests were omitted for most secondary outcomes because the sample size was much smaller than planned, except for length of hospital stay for which a Cox proportional hazards regression was performed (ITT and PP), taking into account censoring (discharge to another hospital) and the competing risk of death.

All analyses were performed using the statistical software R, version 4.3.0.

#### Deviations from the Original Statistical Plan

In some patients, we could not observe the end of the delirium (e.g., due to death, transfer to the general ward with ongoing delirium, or withdrawal of consent) and the delirium duration censored from this point onward. Censoring of the primary endpoint was not considered in the initial analysis plan. Also, the definition of the delirium episode was slightly modified: delirium duration was defined as the start of the treatment with the study medication instead of delirium onset, which could not be adequately timed. The definition of the end of the delirium was modified to the time of the last ICDSC ≥ 4 followed by two subsequent shifts with ICDSC < 4 instead of the end of the shift in which said ICDSC occurred. Due to the much lower sample size than originally planned, secondary outcomes are reported using descriptive statistics only; statistical tests were not performed. Because one patient in the propofol group was transferred to a different hospital with ongoing delirium and four patients died within 28 days, the originally planned number of delirium-free days within 28 days could not be evaluated and imputation was not sensible, as missing was not at random (truncation by death, loss of follow up due to discharge to another hospital). Therefore, we refrained from assessing the difference between the numbers of delirium-free days within 28 days between the two groups but assessed whether another episode of delirium occurred while hospitalized (binary outcome yes/no) for which the truncation by death or discharge was less frequent.

### 2.7. CONSORT Statement

The manuscript was written according to the CONSORT (CONsolidated Standards of Reporting Trials) 2010 guideline. A CONSORT checklist is included within the submission.

## 3. Results

### 3.1. Patients

From April 2019 to March 2022, 752 patients with delirium emerging during their ICU stay were identified. Of those, 714 patients could not be included in the trial because they had one or more exclusion criteria. The most frequent exclusion criteria were “alcohol abuse” (*n* = 124, 17%), “acute cerebrovascular conditions” (*n* = 116, 16%), “status epilepticus” (*n* = 91, 13%), and “delirium prior to ICU admission” (*n* = 80, 11%). A total of 104 screened patients (15%) were screening failures due to other reasons that did not allow an inclusion (Figure 1).

After the Amendment of March 2021 that eliminated “alcohol abuse”, “delirium tremens”, “substance abuse with experience of acute withdrawal”, and “acute cerebrovascular conditions” from the exclusion criteria, a big part of the exclusions were due to “other reasons” (*n* = 59, 34.7%), with an increased amount of patients who were not able to consent in general (e.g., due to dementia, language barrier; 2.2% before and 8.8% of total exclusions after the amendment), or who could not participate due to psychiatric reasons (increase from 0.2% to 4.7%). In addition, there was an increase in cases, in which the treating physicians refused a participation in the study in consideration of the medical situation (increase from 0.9% to 5.3%).

Overall, 38 patients were enrolled and randomized to one of the two treatment groups. In the dexmedetomidine group, four patients did not receive any study medication because of medical reasons (*n* = 2, development of atrioventricular block and worsening of heart index), organizational problems (*n* = 1), or withdrawal of consent (*n* = 1) and were therefore only included in the intention to treat analysis (Figure 1).

The baseline characteristics of the patients are shown in Table 1. In the PP analysis, the median of the SAPS II in the dexmedetomidine group was lower (46, interquartile range [IQR] 41–58.5) compared to the propofol group (57, IQR 47–64.5), as was the number of ventilated patients at baseline (dexmedetomidine group: *n* = 8, 53.3%); propofol group: *n* = 15, 78.9%). The occurrence of the type of delirium among groups was similar: in the intention to treat analysis, nine participants of the dexmedetomidine group were classified as hyperactive and ten as mixed, while in the propofol group seven were classified as hyperactive, and twelve as mixed. In the per protocol analysis there were eight patients with hyperactive delirium and seven patients with mixed delirium in the dexmedetomidine group, while the propofol group did not change.

### 3.2. Trial Interventions

Four patients (two in each group) did not receive the study medication due to rapid recovery. Since this was according to the study protocol, they were included in the per protocol analysis. In the dexmedetomidine group, four patients had a premature study termination, in the propofol group, seven patients did not reach the study end (Figure 1).

### 3.3. Outcomes

#### 3.3.1. Primary Outcome—Delirium Duration

In the intention to treat analysis, the median duration of the delirium episode (timepoint at which 50% of patients had overcome delirium) was shorter in the dexmedetomidine group compared to the propofol group (34 h vs. 66 h). The hazard ratio (HR) of the Cox proportional regression was 1.92 (95% confidence interval [CI] 0.89–4.15, *p*-value 0.097), meaning that at any timepoint the dexmedetomidine group were 1.92 times more likely to have reached the end of the delirium episode than the propofol group. Adjusted for the confounders SAPS II and cardiac surgery (yes/no) the HR was -HR 1.56 (95% CI 0.64–3.75, *p*-value 0.325).

**Figure 2:**
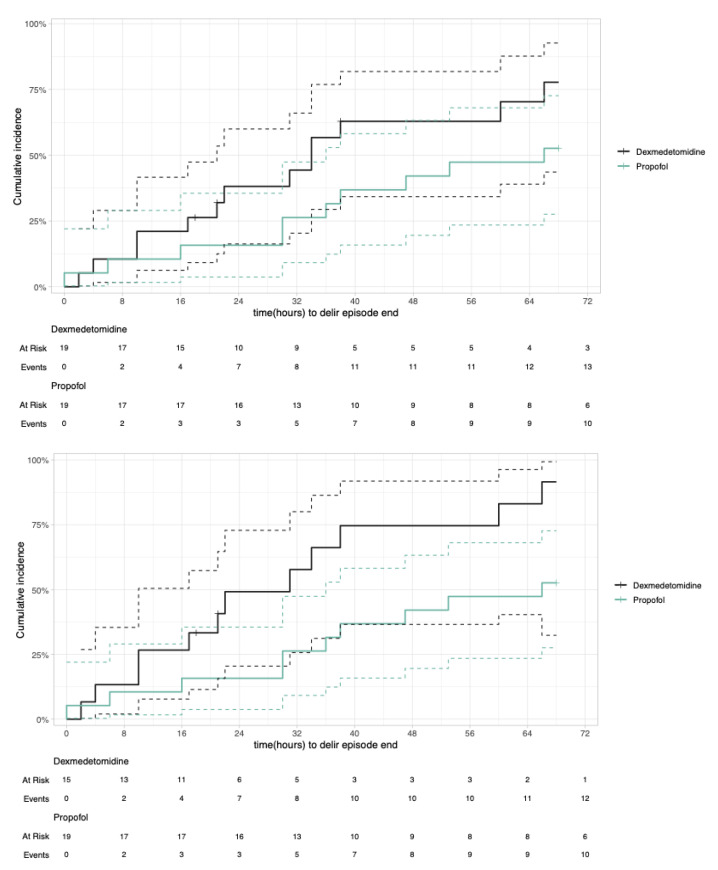
Cumulative incidence plot with competing risk of delirium episode duration. Intention to treat (**top**) and per protocol (**bottom**).

In the per protocol analysis, the median time of the delirium episode was significantly shorter in the dexmedetomidine group compared to the propofol group (31 vs. 66 h, HR 2.95 (95% CI 1.27–6.86, *p*-value 0.012), as shown by the cumulative incidence plot (Figure 2). The HR was similar when adjusted for the confounders SAPS II and cardiac surgery (yes/no; HR 2.91, 95% CI 1.20–7.02, *p*-value 0.018).

#### 3.3.2. Secondary Outcomes

##### Recurrence of Delirium Until 28 Days After ICU Discharge

After discharge to the regular ward delirium relapse within 28 days after ICU discharge was quite frequent. In the per protocol analysis, 11 out of 15 (73%) patients in the dexmedetomidine group and fourteen out of 19 (74%) patients in the propofol group developed another delirium episode after study termination.

##### Death Until Day 28

The number of deaths within 28 days was lower in the dexmedetomidine group compared to the propofol group (intention to treat and per protocol: one death in dexmedetomidine group and five deaths in propofol group; Table 2).

#### 3.3.3. Additional Endpoints

Additional endpoints are listed in Table 2. According to the study protocol, administration of propofol by bolus as a rescue medication was permitted even in the dexmedetomidine group. In total, four patients in the dexmedetomidine group received additional propofol. One patient received two boluses right at the beginning of the first administration of the study medication as well as one bolus the next morning. One patient received propofol intravenously and per bolus for two days additionally to full dosed study medication and other rescue medication. One patient received one bolus of propofol once during the daytime and one patient received a total of three additional bolus of propofol during the administration of the study drug.

### 3.4. Safety End Points

Except for the all-cause deaths, no other severe adverse events occurred. There was no significant increase in abnormalities in oxygen saturation, temperature, or Glasgow coma scale.

**Figure 3:**
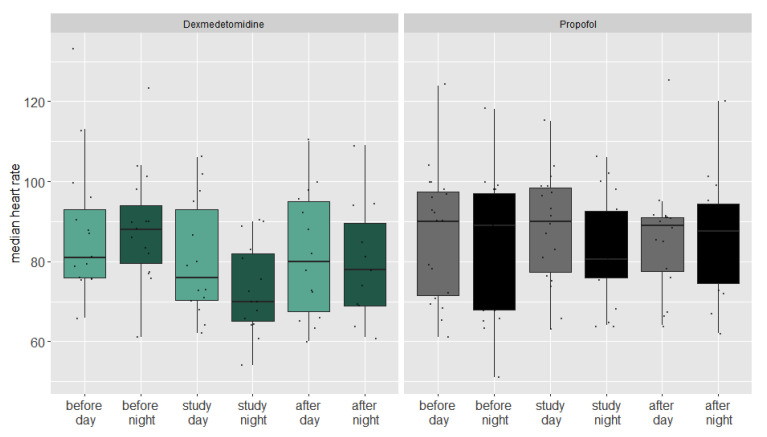
Boxplot of median heart rate during day and night of both groups before during and after the study period.

An analysis of the vital parameters showed that the heart frequency dropped in both groups at night during the administration of the study medication (Figure 3), indicating a relevant antisympathetic effect of both drugs.

In a linear mixed regression model, the heart frequency dropped an estimated 5.9 beats per minute in the propofol group (CI 5.6–6.2, *p* = 0.096). The nightly heart frequency drop in the dexmedetomidine group was 2.9 beats per minute higher compared to the propofol group (CI 2.6–3.2, *p* < 0.001). We could not find a significant nocturnal drop or differences between the groups regarding blood pressure.

## 4. Discussion

In this single-center, randomized controlled trial we found preliminary evidence that adult patients with mixed or hyperactive delirium who received continuous nocturnal infusion of dexmedetomidine had a significantly shorter delirium episode than patients who received nocturnal infusion of propofol. This effect was only found in the per protocol analysis but could not be shown in the intention to treat analysis. In the ITT analysis four more patients were randomized to the dexmedetomidine group, but none was given dexmedetomidine. These four patients had a relatively longer delirium duration compared to most other patients in the same group, which led to a longer median time to delirium end in the dexmedetomidine ITT group. Therefore, the hazard ratio to estimate the difference in the treatment groups was not significant anymore. Such an effect would be expected if the dexmedetomidine had an effect and would shorten delirium duration. Of note, two out of these four patients had undergone heart surgery. But importantly, although the effect was not significant on the 5% level, the estimate still points in the same direction as the per protocol analysis.

We neither found differences between the two groups regarding the median delirium scores (ICDSC) nor for the RASS score during the study period. However, the dexmedetomidine group needed less rescue medication than the patients in the propofol group, which could show an effect on agitation in these patients. Most patients in both groups suffered from further delirium episodes after the study ended.

Although we found a positive effect of dexmedetomidine on the length of delirium, the high prevalence of delirium-relapses in both groups suggests that this treatment could help to terminate an initial episode of a delirium, but it probably does not have any long-time effects on the underlying delirium promoting factors.

The higher mortality of the propofol group after 28 days and in the one-year follow up could either be attributed to chance and/or could be explained by the higher morbidity, as quantified by the SAPS II at baseline in this group [19]. However, to what extent the administration of propofol has contributed to the higher mortality remains unclear. A recently updated meta-analysis seems to show that propofol increases mortality in postoperative and critically ill patients compared to other hypnotic agents [20]. Of note, we did not observe any propofol infusion syndrome in our study patients.

As to our knowledge, this is the first randomized controlled study comparing dexmedetomidine and propofol regarding the treatment of delirium in adults on the ICU. We found two studies that evaluated the use of dexmedetomidine as a treatment of delirium in ventilated ICU patients. The first suggests that the addition of dexmedetomidine to standard therapy in cardiac surgery patients with symptoms of hyperactive delirium leads to less agitation and shorter time to extubation. However, the patients were selected only according to the symptomatic presentation and there was no diagnosis of a delirium [21]. The second study showed that patients with agitated delirium receiving mechanical ventilation had a reduced time to extubation and earlier termination of delirium when they received dexmedetomidine additionally to the standard therapy in comparison with the placebo [22]. The use of dexmedetomidine to prevent delirium in the ICU is better documented. There are several studies that lead to the conclusion that a sedation with dexmedetomidine is favorable over a sedation with propofol to reduce the incidence of delirium in patients undergoing cardiac surgery [23,24,25], mechanically ventilated patients [26] or generally patients in the ICU [17,23]. While using dexmedetomidine, it is important to be aware that Shehabi et al. showed that deep sedation with dexmedetomidine in critically ill patients below a median age of 65 years increased the mortality significantly and should therefore only be considered in elderly patients [27,28].

The study had several strengths, including the prospective, randomized controlled study design. To assess delirium, one of the most frequently used scoring tools was performed at least three times a day. We were able to demonstrate significant differences, although the planned study size could not be reached by far, potentially indicating a substantial effect size.

Our study has several limitations. (I) The study was not blinded out of practicability. (II) Because of a much slower enrolment, the study was terminated prematurely with a much smaller sample size than planned. Therefore, the study is substantially underpowered, challenging the robustness of the results. While the findings are promising, the authors emphasize the exploratory nature of the study due to the premature termination. (III) The results section highlights the statistically significant findings in the per-protocol analysis, but the ITT analysis—being more conservative—did not reach significance. Therefore, the authors point out the risk of overestimating the effects when relying heavily on PP findings. (IV) There was a significant difference in the SAPS II and the number of ventilated patients on admission in the baseline characteristics among groups. (V) According to the study protocol, rescue medication with propofol was allowed in the dexmedetomidine group, too. However, propofol was rarely used in this group. (VI) Most of the patients screened were not eligible for inclusion even after adjustment of the eligibility criteria, questioning the external validity of the study. (VII) This study was conducted in one center.

As stated, the recruitment was much more difficult than expected (see Section Early Termination of the Study Because of Slow Recruitment). Initially, the exclusion criteria were very strict to prevent including patients with an alcohol-associated delirium or patients with acute cerebrovascular conditions. Because many patients in the ICU showed alcohol abuse and/or are suffering from a cerebrovascular disease, we felt that the general ICU population is not well represented and excluded these criteria during the study period. Nevertheless, the enrolment could not be improved to the desired extent. We also had to exclude many patients due to status epileptics/postictal status or could not include them because of psychiatric conditions or the inability to give consent due to dementia in cases where there were not next of kin present.

The difficulties the study team faced during the recruitment process elucidates the challenge of performing RCTs for delirium prevention and therapy. Delirium evolves because of numerous modifiable and non-modifiable risk factors that in many cases cannot be controlled, especially in the ICU. The choice of eligibility criteria to produce real-world evidence for best delirium management may be the biggest challenger and can hamper study conductance overall, as shown by the example of the BaProDex study. Still, those studies are desperately needed to accomplish finding a solution for an unmet medical need: reliable delirium prevention and management strategies.

## 5. Conclusions

Adequately powered randomized controlled trials on delirium in an ICU population are extremely challenging to conduct. Our results suggest that nocturnal infusion of dexmedetomidine should be considered for symptomatic treatment of hyperactive or mixed delirium in the ICU setting and in carefully selected patients.

More studies are needed to assess if a nocturnal sedation with dexmedetomidine may influence delirium due to a restored sleep–wake cycle.

## Figures and Tables

**Table 1:** (**a**,**b**) Demographic and baseline characteristics of study for the full analysis set (as analyzed by ITT and PP).

(a) Baseline Table BaProDex ITT				
			Propofol (*n* = 19)	Dexmedetomidine (*n* = 19)
**Demographics**				
	Males (#, %)		14 (73.7)	13 (68.4)
	Age (median, IQR)		72 [64, 77]	76 [71.5, 78.5]
	Weight (median, IQR)		80 [77, 91.5]	85 [72, 90.5]
	BMI (median, IQR)		28.7 [25.55, 30.45]	27.7 [25.3, 30.1]
**Reason for Admission**				
**Admission Type** (# pat, %)	Medical		8 (42.1)	5 (26.3)
	Scheduled surgical,		2 (10.5)	8 (42.1)
	Unscheduled surgical		9 (47.4)	5 (26.3)
	Heart surgical		4 (21.1)	6 (31.6)
**Admission Information/Scores**				
	SAPS II Score (median, IQR)		57 [47, 64.5]	45 [40.5, 56]
	Initial ICDSC (# pat, %)	4	5 [26.3]	2 [10.5]
		5	2 [10.5]	5 [26.3]
		6	7 [36.8]	4 [21.1]
		7	5 [26.3]	6 [31.6]
	Inital GCS (median, IQR)		11 [8, 14]	13 [11.5, 14]
	Ventilation at admission (# pat, %)		15 (78.9)	9 (47.4)
	Noradrenalin at admission [mg] (median, IQR)		0.1 [0, 0.2]	0 [0, 0.1]
**Past Medical history (# pat, %)**				
	Dementia		0 (0)	1 (5.3)
	ADLQ prior to ICU (median, IQR)		30 [27,32]	30 [25, 31]
	Substance abuse prior to ICU	Tobacco	5 (26.3)	7 (36.8)
		Alcohol	4 (21.1)	3 (15.8)
		Drugs		
	Diabetes		6 (31.6)	8 (42.1)
	Heart rhythm disease		10 (52.6)	8 (42.1)
	Severe or multiple infection		4 (21.1)	1 (5.3)
	Thyroid disease		3 (15.8)	2 (10.5)
	Kidney Disease		4 (21.1)	6 (31.6)
	CVI		3 (15.8)	5 (26.3)
	Intracranial haemorrhage		0 (0)	0 (0)
	Seizures		0 (0)	0 (0)
	M. Parkinsons		0 (0)	1 (5.3)
	Deliriums		0 (0)	0 (0)
	Hypertensions		9 (47.4)	9 (47.4)
	Chronic gastric disease		1 (5.3)	1 (5.3)
	Respiratory distress disease		1 (5.3)	3 (15.8)
	Cancer, ongoing		4 (21.1)	2 (10.5)
	PAD		3 (15.8)	2 (10.5)
**Medication at ICU admission (# pat, %)**				
	ACE-Inhibitoren		2 (10.5)	0 (0)
	Benzodiazepine		0 (0)	0 (0)
	Opiodie		0 (0)	0 (0)
	Narcotics due surgery		3 (15.8)	0 (0)
	Glucocorticoide		1 (5.3)	2 (10.5)
	Antidepressants		0 (0)	1 (5.3)
	NSAID		1 (5.3)	2 (10.5)
	Furosemide		2 (10.5)	2 (10.5)
	Antibiotic		3 (15.8)	3 (15.8)
	Ipratropium bromide		0 (0)	1 (5.3)
	Other		2 (10.5)	2 (10.5)
(**b**) **Baseline Table BaProDex PP**				
			**Propofol (*n* = 19)**	**Dexmedetomidine (*n* = 15)**
**Demographics**				
	Males (#, %)		14 (73.7)	11 (73.3)
	Age (median, IQR)		72 [64, 77]	75 [67, 77]
	Weight (median, IQR)		80 [77, 91.5]	85 [73.5, 90]
	BMI (median, IQR)		28.7 [25.55, 30.45]	27.7 [25.3, 30.1]
**Reason for Admission**				
**Admission Type** (# pat, %)	Medical		8 (42.1)	5 (33.3)
	Scheduled surgical,		2 (10.5)	6 (40)
	Unscheduled surgical		9 (47.4)	4 (26.7)
	Heart surgical		4 (21.1)	4 (26.7)
**Admission Information/Scores**				
	SAPS II Score (median, IQR)		57 [47, 64.5]	46 [41, 58.5]
	Initial ICDSC (# pat, %)	4	5 [26.3]	2 [13.3]
		5	2 [10.5]	3 [20]
		6	7 [36.8]	3 [20]
		7	5 [26.3]	5 [33.3]
	Inital GCS (median, IQR)		11 [8, 14]	13 [11.5, 13.5]
	Ventilation at admission (# pat, %)		15 (78.9)	8 (53.3)
	Noradrenalin at admission [mg] (median, IQR)		0.1 [0, 0.2]	0 [0, 0.1]
**Type of delirium**	hyperactive			
	mixed			
**Past Medical history** (# pat, %)				
	Dementia		0 (0)	1 (6.7)
	ADLQ prior to ICU (median, IQR)		30 [27, 32]	30 [25, 31]
	Substance abuse prior to ICU	Tobacco	5 (26.3)	5 (33.3)
		Alcohol	4 (21.1)	3 (20)
		Drugs		
	Diabetes		6 (31.6)	7 (46.7)
	Heart rhythm disease		10 (52.6)	6 (40)
	Severe or multiple infection		4 (21.1)	1 (6.7)
	Thyroid disease		3 (15.8)	2 (13.3)
	Kidney Disease		4 (21.1)	4 (26.7)
	CVI		3 (15.8)	3 (20)
	Intracranial haemorrhage		0 (0)	0 (0)
	Seizures		0 (0)	0 (0)
	M. Parkinsons		0 (0)	1 (6.7)
	Deliriums		0 (0)	0 (0)
	Hypertensions		9 (47.4)	7 (46.7)
	Chronic gastric disease		1 (5.3)	0 (0)
	Respiratory distress disease		1 (5.3)	3 (20)
	Cancer, ongoing		4 (21.1)	2 (13.3)
	PAD		3 (15.8)	1 (6.7)
**Medication at ICU admission** (# pat, %)				
	ACE-Inhibitoren		2 (10.5)	0 (0)
	Benzodiazepine		0 (0)	0 (0)
	Opiodie		0 (0)	0 (0)
	Narcotics due surgery		3 (15.8)	0 (0)
	Glucocorticoide		1 (5.3)	2 (13.3)
	Antidepressants		0 (0)	1 (6.7)
	NSAID		1 (5.3)	2 (13.3)
	Furosemide		2 (10.5)	2 (13.3)
	Antibiotic		3 (15.8)	3 (20)
	Ipratropium bromide		0 (0)	1 (6.7)
	Other		2 (10.5)	2 (13.3)

Mean ± standard deviation or median [interquartile range] are presented for continuous variables and frequency (percentage) for categorical variables; # = “number of”; BMI, body mass index; SAPS, simplified acute physiology score; ICDSC, Intensive Care Delirium Screening Checklist; GCS, Glasgow Coma Scale; ADLQ, Activities of Daily Living Questionnaire; CVI, cerebrovascular insult; PAD, Peripheral Arterial Disease; ICU, Intensive Care Unit; NSAID, non-steroidal anti-inflammatory drug; pat = patients.

**Table 2:** Secondary outcomes.

	Dexmedetomidine Group *n* = 15	Propofol Group *n* = 19
Death at day 28	1 (7)	5 (26)
Severity of ICU delirium (ICDSC)	4.9 [4.0–5.2]	4.7 [4.0–5.3]
Patients needed ventilation	7 (47)	15 (79)
Ventilation days [days]	0.5 [0–1.8]; total = 30	4 [1–6]; total = 102
Patients received rescue medicaitons	4 (27)	6 (32)
Rescue medication, nr of administrations	11	71
Haloperidol		
Number of patients	5 (33)	9 (47)
Numer of administrations	2 [1–4]	3 [2–9]
Amount of haloperidol [mg]	4 [2–4]; total 18.5	5 [2–9]; total 139
Quetiapine		
Number of patients	8 (53)	11 (58)
Number of administrations	4 [2–7]	8 [2–9]
Amount of oral quetiapine [mg]	44 [12–84]; total 740	80 [74–140]; total 4695
Depth of sedation [RASS]	1.0 [0.0–1.5]	1.0 [0.5–2.0]
Total costs of medication [CHF]	58 [39–242]	16 [6–23]
ICU length of stay [hours]	43 [35–133]	128 (72–330)
Length of hospital stay [days]	10 [6–17]	22 [15–37]
Survival three months after ICU discharge (number of patients died)	2 (13)	7 (37)
Survival twelve months after ICU discharge (number of patients died)	2 (13)	8 (42)
ADLQ		
Patients with baseline	15 (100)	18 (95)
Baseline Score	29.0 [26.1–30.4]	30.4 [27.5–31.9]
Patients participated in 3-month follow-up	11 (73)	9 (47)
Score at 3-month follow-up	29.9 [26.8–30.4]	23.2 [16.0–30.4]
Patients participated in 1-year follow-up	6 (40)	8 (42)
Score at 1-year follow-up	28.3 [23.2–31.2]	21.0 [17.8–30.4]

Per protocol analysis. Median [interquartile range] are presented for continuous variables and frequency (percentage) for categorical variables. Haloperidol, number of administrations/amount of haloperidol: patients with at least one administration of haloperidol; quetiapine, number of administrations/amount of oral quetiapine: patients with at least one administration of quetiapine. ADLQ, Activities of Daily Living Questionnaire; CHF, Swiss francs; ICDSC, Intensive Care Delirium Screening Checklist; ICU, intensive care unit; RASS, Richmond Agitation Sedation Scale.

## Data Availability

The datasets generated and/or analyzed during the current study are not publicly available because ICU-generated data are very sensitive and their containing information could compromise the privacy of the research participants. However, they are available from the sponsor (MS) on reasonable request.

## Supplementary Materials

The following supporting information can be downloaded at: https://www.mdpi.com/article/10.3390/jcm14124348/s1, Figure S1: Graphical algorithm of the Standard Operating Procedure of the University Hospital Basel for treatment of hyperactive delirium; Figure S2: Timeline of screening-process showing the screened, enrolled and dropped out patients over time; Figure S3: Overview of delirium, censoring and death during hospital stay of included patients before, during and after study period; Table S1: Time and reason of death of all patients who died between inclusion date and one year follow up.
