# Supplementary material for: Comparison of Propofol and Dexmedetomidine Infused Overnight to Treat Hyperactive and Mixed ICU Delirium: A Prospective Randomised Controlled Clinical Trial
Source: J Clin Med. 2025 Jun 18;14(12):4348. doi: 10.3390/jcm14124348 (PMC12194003; doi:10.3390/jcm14124348)
Supplement: Supplementary file 1 [file jcm-14-04348-s001.zip › jcm-3563997-supplementary.pdf]

BaProDex – SUPPLEMENT

Supp Figure S1: Graphical algorithm of the Standard Operating Procedure of the University Hospital Basel for treatment of hyperactive delirium.

Supp Figure S2: Timeline of screening-process showing the screened, enrolled and dropped out patients over time.

Supp Figure S3: Overview of delirium, censoring and death during hospital stay of included patients before, during and after study period.

Supplementary Figure S1

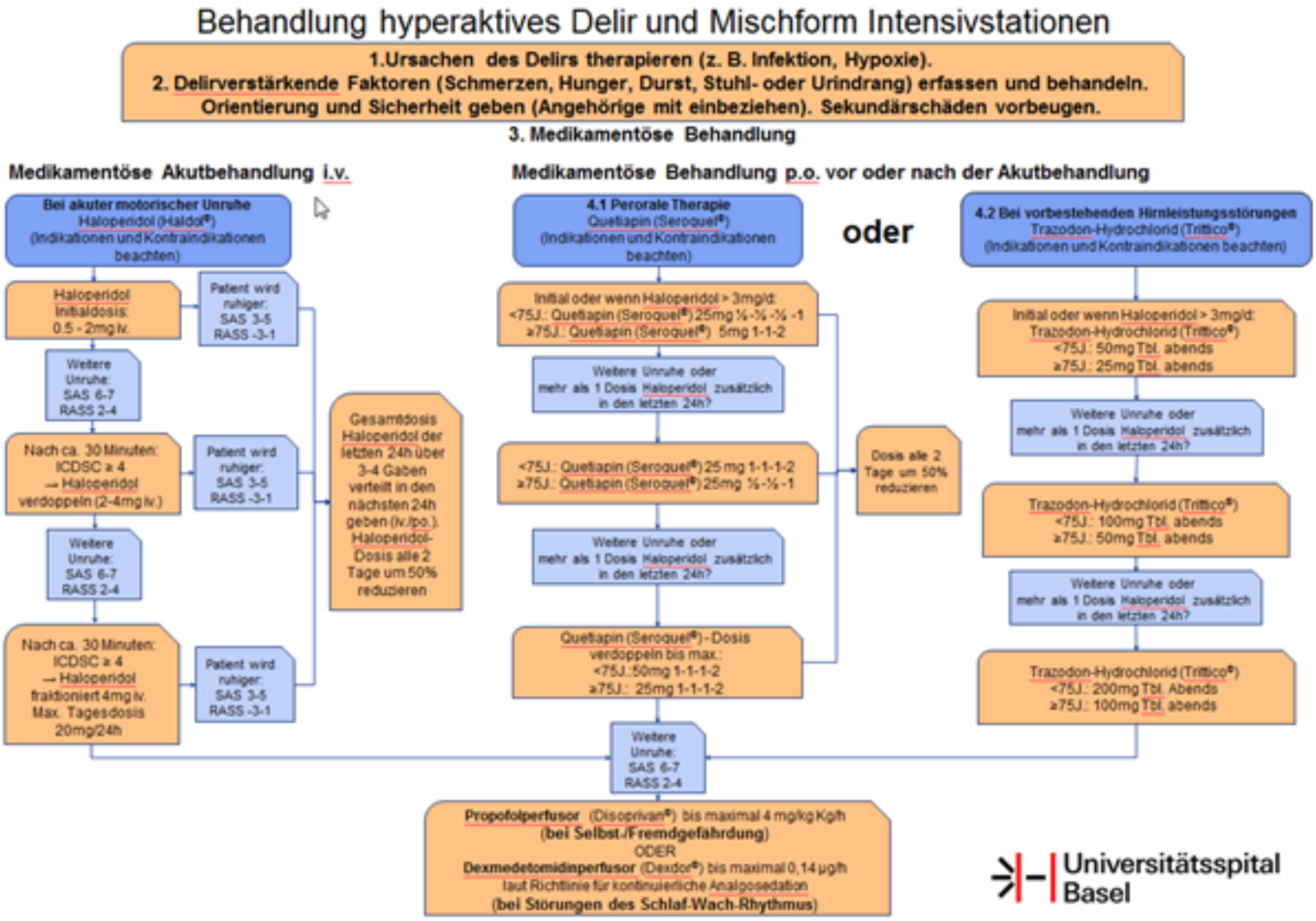

Supplementary Figure S2

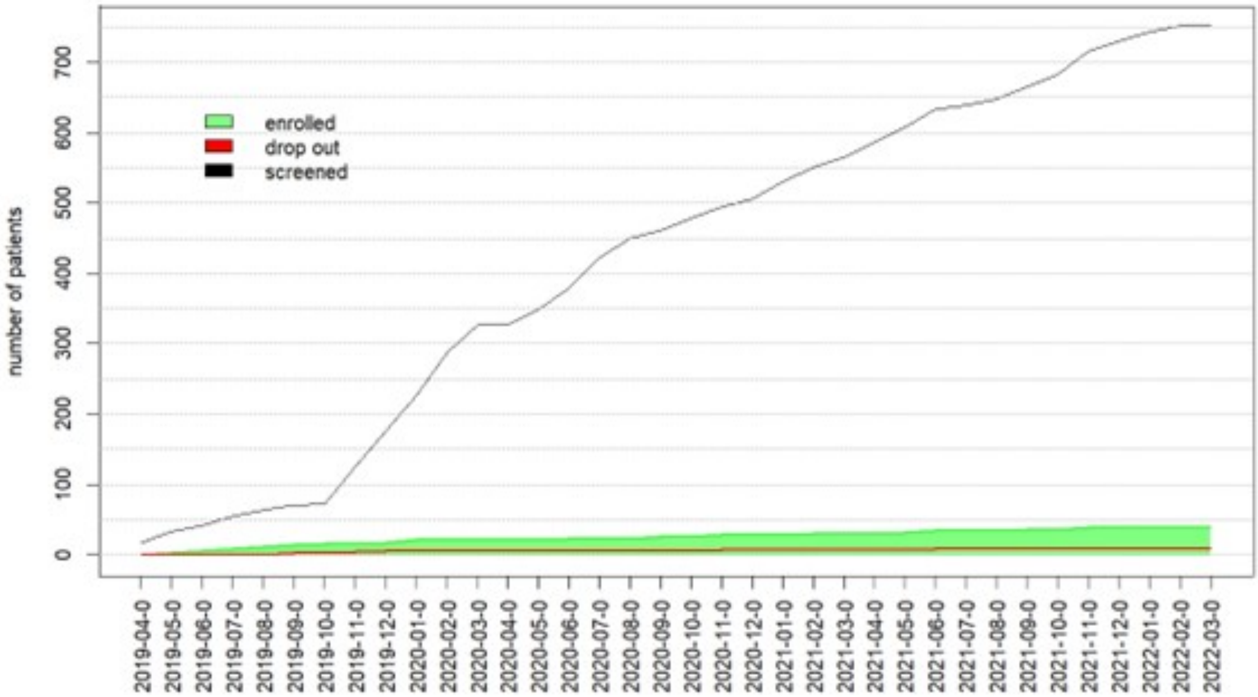

Supplementary Figure S3

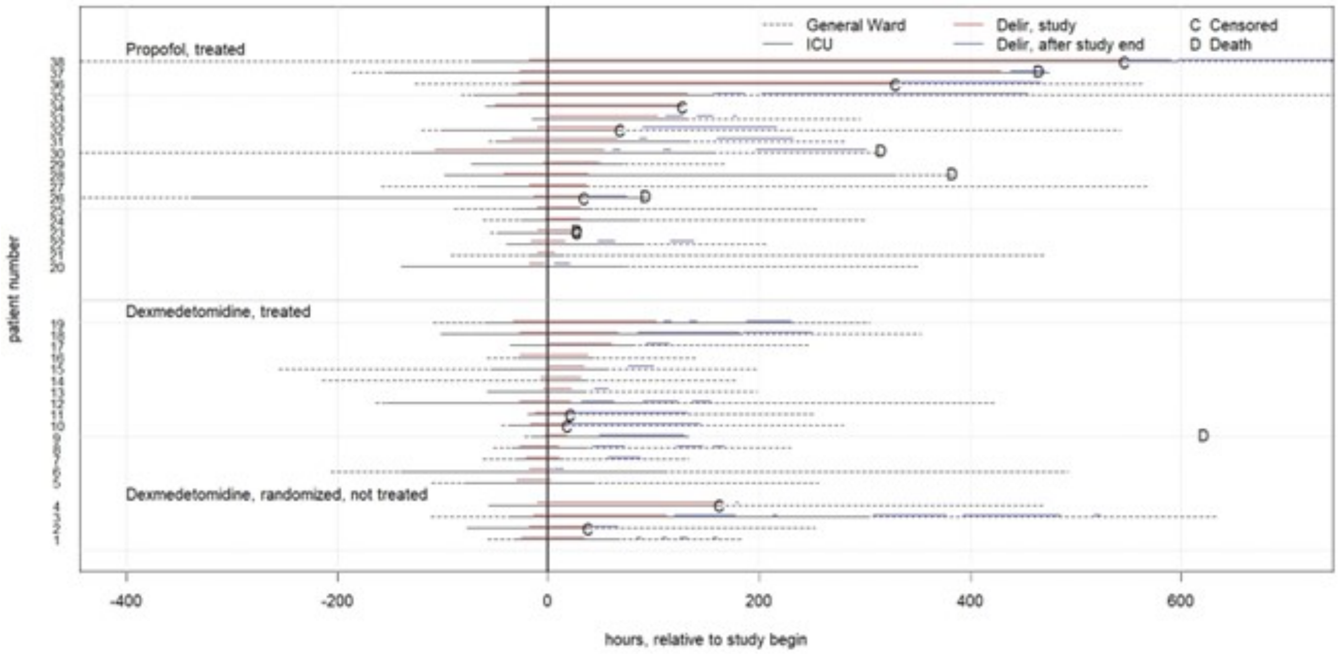

Supplementary Table S1. **Time and reason of death of all patients who died between inclusion date and one year follow up.**

**Group**	**Patient Number**	**Time of death**	**reason of death**
propofol	23	0	mesenterial ischemia after thoracic endovascular aortic repair because of aortic dissection type B
propofol	26	2	Covid-19
propofol	37	3	multiorgan failure because of sepsis of multifactorial aetiology (peritonitis, pneumonia, obstructive pyelonephritis)
propofol	30	11	multiorgan failure because of sepsis after postoperative liver abscess
propofol	28	14	sepsis after intestinal perforation because of rectal cancer
dexmedetomidine	9	20	tracheobronchitis, hospital acquired pneumonia
propofol	21	36	deceased, out of hospital
propofol	38	74	deceased, out of hospital
dexmedetomidine	11	102	deceased, out of hospital
propofol	35	135	deceased, out of hospital
dexmedetomidine	-	< 1 year	deceased, out of hospital
dexmedetomidine	-	< 1 year	deceased, out of hospital

Patient Number: The patient number corresponds to the number shown in Supplementary Figure 3. Time of death: day after study end; patients with time of death <1 year were included in intention to treat analysis only
